# Pernicious Anemia with Autoimmune Hemolytic Anemia: A Case Report and Literature Review

**DOI:** 10.1155/2016/7231503

**Published:** 2016-07-31

**Authors:** Sri Lakshmi Hyndavi Yeruva, Raj Pal Manchandani, Patricia Oneal

**Affiliations:** Division of Hematology and Oncology, Department of Internal Medicine, Howard University Hospital, Washington, DC 20060, USA

## Abstract

Pernicious anemia is a common cause of vitamin B12 deficiency. Here, we discuss a case of a young woman who presented with severe anemia along with a history of iron deficiency anemia. After a review of her clinical presentation and laboratory data, we identified an autoimmune hemolytic anemia and a concomitant pernicious anemia. The concurrence of both these hematological diagnoses in a patient is rare.

## 1. Introduction

Vitamin B12 deficiency is common in the United States and pernicious anemia is one of the most common causes of vitamin B12 deficiency throughout the world [1]. Patients with vitamin B12 deficiency usually present with hematologic and neurologic findings. The hematologic manifestations can range from megaloblastic anemia to pancytopenia and can be life threatening in approximately 10% of patients [2]. In this paper, we present a young female with a history of iron deficiency anemia who came with severe anemia secondary to autoimmune hemolytic anemia (AIHA) and a vitamin B12 deficiency caused by a deficiency in intrinsic factor leading to pernicious anemia.

## 2. Case Report

A 22-year-old African American female with past medical history of hypertension and iron deficiency anemia presented with generalized fatigue and new onset dyspnea on exertion of three-week duration. She started having heavy menstrual bleed requiring blood transfusions in last six months and recent transfusion of four units of packed red blood cells (PRBCs) was one month ago at an outside facility. Her last menstrual cycle was also a month ago. She denied any history of autoimmune diseases or history of miscarriages. She is sexually active with no history of sexually transmitted diseases. Her only home medications included iron supplements and oral contraceptive pills.

On admission, her vitals and physical examination were normal except for a heart rate of 132 beats/minute, pale conjunctiva with an icteric sclera, and morbid obesity. She did not have any organomegaly and neurologic examination was normal. Further review of labs showed hemoglobin and hematocrit of 4.8 g/dL (normal range: 12.1–15.9) and 13.4% (normal range: 34.3–46.6), respectively, with platelets of 91,000 (normal range: 177000–406000), MCV 87.7 fL (normal range: 77.8–98), RDW 14.4%, absolute reticulocyte count of 97,000, and reticulocyte percent of 7.8. Additional labs included serum iron 273 mc/dL (normal range: 55–185), ferritin 500 ng/mL (normal range: 20–400), transferrin of 234 mg/dL (normal range: 192–382), haptoglobin 2 mg/dL (normal range: 36–195), and LDH 1868 IU/L (normal range: 0–250). Renal, coagulation, and hepatic function tests were normal except for elevated total bilirubin of 1.5 mg/dL (normal range: 0.2–1.2). The peripheral smear showed marked anisocytosis, hypochromasia with poikilocytosis, many macrocytes, microcytes, and few schistocytes. Given her recent blood transfusion, we tried to determine if the hemolytic process was secondary to AIHA versus delayed hemolytic transfusion reaction. Her results showed no alloantibodies; however, a direct antiglobin test (Coombs) with IgG was found to be positive which was suggestive of AIHA. Further anemia workup also revealed a vitamin B12 deficiency at 60 pg/mL (250–950), folate 7.26 ng/mL (5.9–24.8), and normal thyroid profile. Homocysteine and methylmalonic acid levels were elevated at 22.4 *μ*mol/L (<10.4) and 2300 nmol/L (87–813), respectively. These laboratory results confirmed a vitamin B12 deficiency. The etiology of the vitamin B12 deficiency was then ascertained with presence of intrinsic factor blocking and anti-parietal cell antibodies as lack of intrinsic factor and loss of parietal cells, resulting in pernicious anemia. Autoimmune workup with anti-centromere antibody, cyclic citrullinated peptide, ANA levels, rheumatoid factor along with serum immunoglobulins, and complements were negative. Her hepatitis panel showed immunity to hepatitis B and nonreactivity to hepatitis C and HIV.

After receiving two units of PRBCs for her symptomatic anemia, she was started on prednisone 100 mg (1 mg/kg of body weight) daily for the treatment of AIHA. Meanwhile, given her concomitant vitamin B12 deficiency, she was also initiated on daily parenteral vitamin B12 supplementation along with iron and folic acid supplementation. With these treatments, her hemoglobin and hematocrit levels improved to 7.1 g/dL and 20.9% with a good reticulocyte response of 345,000 over a course of five days of treatment. Steroids were then decreased to prednisone 40 mg and patient was discharged home.

Outpatient upper GI endoscopy was performed which showed patchy area of nonerosive gastritis in the antrum and atrophic mucosa in the body of the stomach. Biopsy of the atrophic mucosa revealed chronic active atrophic gastritis with intestinal metaplasia and immunostaining for* Helicobacter pylori* was negative. These findings were consistent with pernicious anemia.

On her subsequent clinic visits, we continued her on prednisone 40 mg daily and monthly subcutaneous vitamin B12 supplementation until her hemoglobin and hematocrit stabilized. After the direct Coombs test became negative at six months, with a stable hemoglobin more than 10 g/dL, normal LDH, and reticulocyte count < 100,000, the steroids were gradually tapered off over two months while she was continued on subcutaneous vitamin B12 supplements.

Her laboratory data from most recent follow-up in clinic, which was two years after her initial presentation, was hemoglobin and hematocrit of 12.4 g/dL and 38%, respectively, MCV 88 fL with a reticulocyte percent of 3.5.

## 3. Discussion

Vitamin B12 deficiency varies and increases with age. Prevalence of vitamin B12 deficiency is 3% in patients aged 20 to 39 years [3, 4]. Pernicious anemia is an autoimmune disease caused by vitamin B12 deficiency due to atrophic gastritis or loss of parietal cells or lack of intrinsic factor. A diagnosis of pernicious anemia is made when patients have a low vitamin B12 level and positive intrinsic factor antibody or parietal cell antibody or low vitamin B12 level and presence of atrophic gastric mucosa, establishing the autoimmune nature of the disease and presence of vitamin B12 deficiency. Given the difficulty with accurate diagnosing of vitamin B12 deficiency on the currently available methods, patients with low normal vitamin B12 levels could still be deficient in it and in these instances, a diagnosis is made by elevated methylmalonic acid and homocysteine levels [5].

Vitamin B12 deficiency can present with a hemolytic picture in 1.5% of patients with elevated LDH, low haptoglobin, and elevated indirect bilirubin mostly due to ineffective erythropoiesis and intramedullary destruction [2, 4]. Managing our patient was challenging as she presented with a severe normocytic anemia and hemolytic picture, none of which were suggesting vitamin B12 deficiency. Further evaluation for causes of anemia revealed a diagnosis of vitamin B12 deficiency due to deficiency of intrinsic factor and subsequently a diagnosis of pernicious anemia was made. As no alloantibodies were detected on further workup, a delayed hemolytic transfusion reaction as a cause for her hemolysis was ruled out.

As the management of anemia due to pernicious anemia with hemolysis is different from patients presenting with autoimmune hemolytic anemia (AIHA) with a positive direct Coombs test, differentiating these two conditions early on is important. Although it has been reported that patients with pernicious anemia can transiently have a positive Coombs test which becomes negative on treatment with vitamin B12 supplementation, patients with true AIHA would not respond to vitamin B12 supplementation, unless they are treated with steroids [6].

The autoimmune nature of the pernicious anemia is not fully understood but patients with this disease can have higher incidence of other autoimmune diseases, which might be due to a shared susceptibility in some individuals [5]. These two autoimmune mediated conditions (pernicious anemia and AIHA) can present concomitantly as in our patient or can be preceded by one another. The presence of autoimmune hemolytic anemia with a positive antiglobin test is not a common finding. In a study by Pirofsky and Vaughn, it was found that, among 103 patients with pernicious anemia, only 5 patients had positive antiglobin test [7]. In another study involving 865 patients with autoimmune hemolytic anemia, only three patients with pernicious anemia were positive for direct Coombs test [8].

A further literature search was done of published case reports and case series on autoimmune hemolytic anemia with a positive direct Coombs test in patients with pernicious anemia. As seen in Table 1, this association was noted mostly in women who were middle aged with other autoimmune conditions.

## 4. Conclusion

Though AIHA and pernicious anemia are not common, clinicians should have a high index of suspicion when patients present with hemolytic picture and severe megaloblastic anemia, as a careful and systematic approach to diagnose the etiology of anemia can prevent patients from undergoing unnecessary procedures and treatments.

## Figures and Tables

**Table 1:** Reported cases of pernicious anemia with autoimmune hemolytic anemia.

Serial number	Author	Year	Number of cases	Age	Sex	Other autoimmune diseases
1	Selwyn and Alexander [9]	1951	1	38	M	None
2	Rubio Jr. and Burgin [10]	1957	1	64	F	None
3	Willoughby et al. [11]	1961	1	60	F	None
4	Forshaw and Harwood [12]	1965	5	N/A	N/A	N/A
5	Pirofsky and Vaughn [7]	1968	5	77 60 67 60 75	M M F F M	None None None None SLE
6	Salvidio et al. [13]	1975	1	56	F	None
7	Nel [14]	1983	1	16	F	Primary acquired hypogammaglobulinemia
8	Baba and Maharaj [15]	1988	1	71	F	Hypocalcemia
9	Pelosio et al. [16]	1989	1	61	F	Vitiligo
10	Feld et al. [17]	1989	1	66	F	SLE, Sjögren, Hashimoto's thyroiditis
11	Rabinowitz et al. [6]	1990	1	61	F	None
12	Zafad et al. [18]	2007	1	16	M	Alopecia areata
13	Vucelic et al. [19]	2008	1	55	F	Rheumatoid arthritis
14	Aziz et al. [20]	2010	1	80	F	None
